# Development and Validation of a Plaque Assay to Determine the Titer of a Recombinant Live-Attenuated Viral Vaccine for SARS-CoV-2

**DOI:** 10.3390/vaccines12040374

**Published:** 2024-04-01

**Authors:** Einat Toister, Lilach Cherry, Edith Lupu, Arik Monash, Eyal Dor, Lilach Levin, Meni Girshengorn, Niva Natan, Shira Chapman, Shlomo Shmaya, Eyal Epstein, Yaakov Adar, Ran Zichel, Yakir Ophir, Eran Diamant

**Affiliations:** 1 Department of Biotechnology, Israel Institute for Biological Research, Ness Ziona 7410001, Israel; einatt@iibr.gov.il (E.T.); lilachc@iibr.gov.il (L.C.); edithl@iibr.gov.il (E.L.); arikmo@iibr.gov.il (A.M.); eyalo@iibr.gov.il (E.D.); menig@iibr.gov.il (M.G.); nivan@iibr.gov.il (N.N.); eyale@iibr.gov.il (E.E.); yaakova@iibr.gov.il (Y.A.); ranz@iibr.gov.il (R.Z.); 2 Department of Infectious Diseases, Israel Institute for Biological Research, Ness Ziona 7410001, Israel; shlomosh@iibr.gov.il; 3 Department of Pharmacology, Israel Institute for Biological Research, Ness Ziona 7410001, Israel; shirac@iibr.gov.il; 4 Department of Microbiology, Cornell University, Ithaca, NY 14850, USA

**Keywords:** PFU, plaque assay, validation, SARS-CoV-2, COVID-19, vaccine

## Abstract

The COVID-19 pandemic, caused by severe acute respiratory syndrome coronavirus 2 (SARS-CoV-2), has resulted in more than seven million deaths worldwide. To reduce viral spread, the Israel Institute for Biological Research (IIBR) developed and produced a new rVSV-SARS-CoV-2-S vaccine candidate (BriLife^®^) based on a platform of a genetically engineered vesicular stomatitis virus (VSV) vector that expresses the spike protein of SARS-CoV-2 instead of the VSV-G protein on the virus surface. Quantifying the virus titer to evaluate vaccine potency requires a reliable validated assay that meets all the stringent pharmacopeial requirements of a bioanalytical method. Here, for the first time, we present the development and extensive validation of a quantitative plaque assay using Vero E6 cells for the determination of the concentration of the rVSV-SARS-CoV-2-S viral vector. Three different vaccine preparations with varying titers (DP_low, DP_high, and QC sample) were tested according to a strict validation protocol. The newly developed plaque assay was found to be highly specific, accurate, precise, and robust. The mean deviations from the predetermined titers for the DP_low, DP_high, and QC preparations were 0.01, 0.02, and 0.09 log_10_, respectively. Moreover, the mean %CV values for intra-assay precision were 18.7%, 12.0%, and 6.0%, respectively. The virus titers did not deviate from the established values between cell passages 5 and 19, and no correlation was found between titer and passage. The validation results presented herein indicate that the newly developed plaque assay can be used to determine the concentration of the BriLife^®^ vaccine, suggesting that the current protocol is a reliable methodology for validating plaque assays for other viral vaccines.

## 1. Introduction

SARS-CoV-2, the virus responsible for the global coronavirus pandemic, has infected more than 750 million people worldwide and claimed the lives of more than seven million individuals [1]. Much effort has been expended in both developing vaccines and finding drugs to neutralize viruses and treat direct and indirect damage.

Most vaccines are mainly aimed at creating neutralizing antibodies that prevent the binding of the receptor-binding domain (RBD), which is part of the viral spike protein, to the membrane protein angiotensin-converting enzyme 2 (ACE2) on the surfaces of the target cells.

The Israel Institute for Biological Research (IIBR) developed and manufactured a new vaccine candidate, named “Brilife^®^”, to fight infection with and the spread of SARS-CoV-2 throughout the population in Israel [2,3,4,5,6]. The approach adopted by the IIBR was based on one found to be effective against the Ebola virus in Africa [7,8]. This approach involves the genetic engineering of a vesicular stomatitis virus (VSV) vector to express the spike protein from SARS-CoV-2 instead of the VSV-G protein on the virus surface. Following the cloning of the SARS-CoV-2 spike gene into the VSV plasmid instead of the G open reading frame, the complete plasmid sequence was confirmed via next-generation sequencing (NGS). Upon infection with this construct (rVSV-SARS-CoV-2-S), the expressed spike protein induces both neutralizing antibodies and a T-cell immune response [6]. VSV is a negative-sense RNA virus of the Rhabdoviridae family that contains an RNA genome of approximately 11 kb that encodes five major proteins. Moreover, VSV has been established as a robust vaccine vector backbone for infectious diseases for more than a decade and is considered to be an ideal vaccine vector candidate for pathogen outbreaks [9]. Given the urgency of finding a solution to the COVID-19 outbreak, the IIBR has adopted this development approach.

Virus quantification in various control stages throughout production in process control (IPC), as well as in the final stages (the drug substance (DS) and drug product (DP) stages), was performed using the traditional standard plaque assay, a widely used and reliable technique for quantifying virus titer, i.e., the concentration of the infectious virus [10]. Importantly, during clinical trials, vaccine doses are determined according to the validated plaque assay results.

The assay is based on the formation of a destruction center by one virus in a confluent layer of cells; each infectious viral particle produces a plaque—a circular zone of infected cells surrounded by uninfected cells that becomes large enough to be visible to the naked eye. The PFU/mL result represents the concentration of infective particles within the sample based on the assumption that each plaque formed is representative of one infective virus particle. This method allows the quantification of the viral titer throughout the IPC steps, including the DS and DP. Most importantly, the plaque assay may serve as a validated method for determining vaccine titers as a basis for determining the potency of the IIBR virus-based vaccine.

In this study, following the establishment of a newly developed plaque assay, a validation protocol was designed to provide sufficient evidence that the analytical procedure could meet its objectives and would be suitable for viral analysis in DS and DP solutions; both contain the same matrix and were, therefore, validated in the work shown here.

Assay validation was performed according to the requirements of the European Pharmacopoeia (EP) for PFU determination for the smallpox vaccine (live) [11] and following the International Council for Harmonisation (ICH) Q2 (R1) concerning the validation of analytical procedures [12] under Good Manufacturing Practice (GMP) conditions. Extensive validation of the developed method demonstrated suitable specificity, linearity, precision, accuracy and robustness for measuring the concentrations of DSs and DPs of the vaccine. Notably, given the scarcity of publications in the field of plaque assay validation, the current protocol has the potential to become a dependable methodology for the validation of plaque assays for other viral vaccines, as well as a practical guide for fulfilling regulators’ requirements for this important assay.

## 2. Materials and Methods

### 2.1. Cells

Vero E6 cells were obtained from the American Cell Bank at the ATCC (C1008; lot number: 70034202). Pooled Master and Working Cell Banks (MCB and WCB, respectively) were prepared and characterized at the IIBR Cell Culture Research Unit. The MCB and WCB vials were stored in the vapor phase of liquid nitrogen tanks according to the internal procedures of the IIBR. During the prevalidation stage of the assay, the process of preparing the plates with the cells was optimized, and the process included the number of cells seeded in each well, the seeding volume, the incubation period prior to virus addition and the maximum number of passages allowed (<20). In total, 3 mL of suspension containing 700,000 cells in Dulbecco’s modified Eagle’s medium (DMEM; Sartorius, Beit Haemek, Israel) supplemented with 10% fetal bovine serum (Sartorius, Beit Haemek, Israel), 2 mM of L-glutamine (Sartorius, Beit Haemek, Israel), and 1% nonessential amino acids (Sartorius, Beit Haemek, Israel) was seeded in each well. The plates were then incubated in a 5% CO_2_ incubator for 24 h at 37 °C. Prior to each assay, the confluency of the cells was verified under an inverted microscope (Eclipse Ts2R, Nikon Instruments Inc., Tokyo, Japan).

### 2.2. Plaque Assay

**Figure 1:**
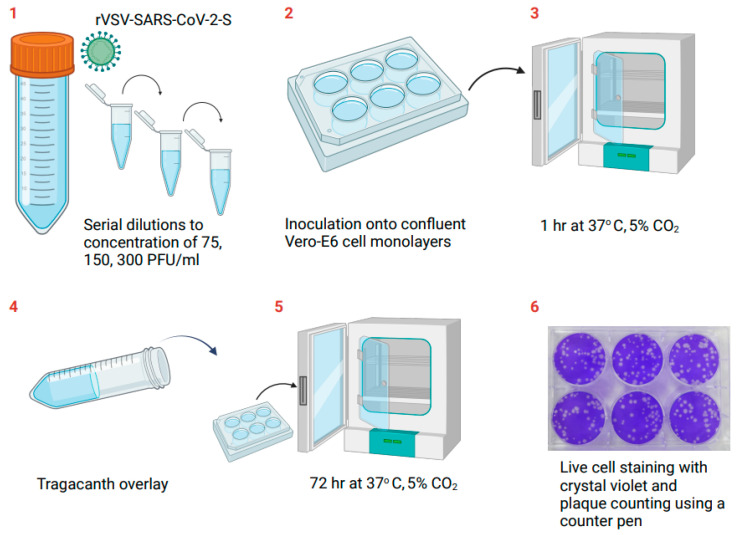
Schematic description of the test steps for determining the rVSV-SARS-CoV-2-S concentration in Vero E6 cell culture using the plaque assay. Adapted from illustrations created via BioRender.com (2020). Retrieved from https://app.biorender.com/biorender-templates, accessed on 16 February 2024.

Tenfold dilutions of the rVSV-SARS-CoV-2-S sample were prepared in minimal essential medium (MEM; Sartorius, Beit Haemek, Israel). Then, an additional dilution was conducted prior to final three twofold dilutions to concentrations of 75, 150, and 300 PFU/mL in 0.2 mL (15, 30, and 60 PFU/well, respectively). The accepted range was set to 10–80 PFU/well. A volume of 0.2 mL of each of the three final dilutions was inoculated onto confluent Vero-E6 cell monolayers in 6-well tissue culture plates in six replicates. After an incubation period of one hour at 37 °C and 5% CO_2_, to allow the virus to penetrate the cells, the monolayers were covered with tragacanth (Merck, Israel) gum solution-based medium (MEM), supplemented with 0.4% tragacanth (Merck, Israel), 2% fetal bovine serum (Sartorius, Beit Haemek, Israel), 2 mM of L-glutamine (Sartorius, Beit Haemek, Israel), 1% nonessential amino acids (Sartorius, Beit Haemek, Israel), and 0.15% sodium bicarbonate (Sartorius, Beit Haemek, Israel). The tragacanth medium forms a semigel overlay and restricts the spread of new viral progenies to neighboring cells. The plates were incubated for 72 h at 37 °C and 5% CO_2_. Consequently, each infectious viral particle produced a plaque. At the end of the incubation, the tragacanth medium was aspirated, and 1 mL of crystal violet solution (0.1% crystal violet dye (*w*/*v*) in ethanol/water (20/80), Sartorius, Beit Haemek, Israel) was added to the wells to stain the living cells and enhance the contrast between the plaques and the surrounding living cells. The plaques were then counted using a counter pen (hand-held colony counter, SP Bel-Art). Wells containing either less than 10 PFU or more than 80 PFU were omitted from the calculation of the mean. The titer in PFU/mL was calculated by multiplying the mean PFU/well by the dilution factor and by 5 (to normalize the inoculum volume from the actual 0.2 mL to a calculated 1 mL). A schematic description of the plaque formation assay in Vero E6 cell culture is presented in Figure 1.

### 2.3. Method Validation

Validation was performed with three rVSV-SARS-CoV-2-S samples, which served as controls with known concentrations to confirm the results. (1) A QC sample was a DS preparation with a known concentration of 6.50 × 10^7^ PFU/mL within an acceptance range of 2.06 × 10^7^–2.06 × 10^8^ PFU/mL based on the values determined during prevalidation and given a deviation of ±0.5 log_10_ PFU/mL. The DS was prepared by diluting the starting material, i.e., the cell harvest following downstream purification steps, in a buffer formulation containing Tris(hydroxymethyl)aminomethane (Tris) (Merck, Darmstadt, Germany), recombinant human serum albumin (rHSA, Invitria, Junction city, KS, USA), NaCl (Merck, Darmstadt, Germany), and trehalose (Pfanstiehl, Waukegan, IL, USA). (2) A DP_low sample was obtained from a DP preparation with a known concentration of 1.17 × 10^7^ PFU/mL, within an acceptance range of 3.70 × 10^6^–3.70 × 10^7^ PFU/mL, based on the values determined during prevalidation and given a deviation of ±0.5 log_10_ PFU/mL. (3) A DP_high sample was obtained from a DP preparation with a known concentration of 1.60 × 10^8^ PFU/mL, within an acceptance range of 5.06 × 10^7^–5.06 × 10^8^ PFU/mL, based on the values determined during prevalidation and given a deviation of ±0.5 log_10_ PFU/mL.

DP_low and DP_high samples were prepared by diluting different DS batches (both of which differed from the QC sample) in a buffer formulation containing Tris, rHSA, NaCl, and trehalose. All the samples were divided into aliquots and stored in a −70 °C freezer. In each test, a single-use thawed ampoule was used.

The assays used during validation were performed according to a formal standard operation procedure (SOP), making the necessary changes to each of the designated assays in accordance with the pharmacopeial requirements [11]. For each of the assays performed, the reference sample was determined in three repetitions of a single dilution (i.e., a plate with 6 replicates per repetition), which were sampled three times from a single tube (6 samples per plate, a total of 3 plates for a single sample, and a total of 18 repetitions). The assay was used to determine the titer from three vials by a single operator, with each vial being tested in three dilutions and each dilution being tested in six repetitions per plate.

The assays were performed as follows: For the DP_low sample, two assays were performed simultaneously on three dates (at each date) by two analysts. Three analysts performed additional assays simultaneously. Accordingly, nine assays were performed. The tests were performed on cells from different growth cycles from the beginning and end of the range approved for use (20 growth cycles). For DP_high, two assays were performed on two different dates in parallel by two analysts from three different vials. Accordingly, four assays were performed to determine the titer.

The samples were mixed for seeding in TC-6 wells to approximate concentrations of 75, 150, and 300 PFU/mL so that approximately 15, 30, and 60 PFU/well were obtained by seeding 0.2 mL per well, respectively. The calculation of the amount of virus in the sample examined was based only on wells in which 10–80 PFU/well were obtained so that individual plaques could be counted with certainty.

The titer was calculated from three different thawed frozen vials, as described above. The dilution of each vial was determined as specified in the SOP, with each dilution seeded in a plate at six repetitions (wells) per dilution for each vial so that each operator evaluated three plates (P1, P2, and P3) per vial, with a total of 18 repetitions for three dilutions and a total of 54 repetitions per date.

Each assay involved QC determinations on three independent repetitions of a single dilution, each with six repetitions (wells) in the plate; a total of three plates (P1, P2, and P3) were used for each assay.

The calculation of the virus concentration in the DP samples was based on an average of the three frozen-thawed vials. For each vial, the average was calculated from at least eight repetitions of all dilutions. According to the pharmacopoeia, the following conditions must be met for the test result to be considered acceptable:

1. The confidence interval (*p* = 0.95) (95% CI) for the combined virus concentration (in three vials of the sample tested) was not greater than ±0.5 log_10_ PFU/mL;
2. The confidence interval (*p* = 0.95) (95% CI) of the estimated virus concentration of the reference preparation (QC sample) for the three replicates was not greater than ±0.5 log_10_ PFU/mL;
3. The virus concentration of the reference sample (QC sample) in the test differed by less than 0.5 log_10_ PFU/mL from the established value.

As a negative control, two wells containing a cell monolayer without virus were seeded, and the test result was considered acceptable when the monolayer was undamaged and no plaques were visible.

All plaque assays were conducted in compliance with GMP regulations in a GMP facility. The plaques were counted using a hand-held colony counter by a certified analyst. The plaque counts were manually copied to a paper batch record. Concomitantly, the data were transferred to secured Microsoft Excel sheets that had been created and validated to include protected algorithms for calculating means and standard deviations. As part of the validation protocol, further analyses to compare results between assays and calculate regression lines and ANOVA were conducted with GraphPad Prism 5. The figures were created using GraphPad Prism 5.

## 3. Results

Following the development and establishment of the plaque assay, a validation protocol was carried out to demonstrate that the analytical procedure was suitable for quantifying the titer of rVSV-SARS-CoV-2-S. Validation was performed according to the requirements of the ICH Q2 (R1) concerning the validation of analytical procedures under strict GMP regulations. The certified validation protocol included measurements of specificity, linearity, range, accuracy, precision, detection and quantitation limits, and robustness.

### 3.1. Specificity

The specificity of the plaque assay was evaluated by conducting an identification test. For this purpose, two virus-negative matrix samples were prepared by spiking either growth medium (FLEX20 [Sartorius, Israel]) with known process- and product-related impurities, including Vero host cell proteins (HCPs), Vero DNA, and denarase, at appropriate concentrations or with equilibration buffer containing Tris, HSA, NaCl, and trehalose. This enabled the simulation of either IPC (vir_neg-IPC-MTRX) or DS samples (vir_neg-DS-MTRX) without rVSV-SARS-CoV-2-S, respectively. Additionally, virus-positive samples (vir_pos-IPC-MTRX and vir_pos-DS-MTRX) were prepared by further spiking each of the virus-negative matrices with a calibrated DP_low sample (1:10). The discrimination of the assay was confirmed by obtaining positive, anticipated viral titers of 1.0 × 10^7^ PFU/mL and 1.2 × 10^7^ PFU/mL from the vir_pos-IPC-MTRX and vir_pos-DS-MTRX samples, respectively (both containing the virus, i.e., the tested analyte), coupled with negative results from the vir_neg-IPC-MTRX and vir_neg-DS-MTRX samples, which did not contain the virus. The accuracy of the assay can also rule out the bias of the results by impurities and excipients. Notably, the levels of impurities in the simulated MTRX samples resembled those of the highest concentrations measured during the first steps of the downstream process. Hence, the tested specificity conditions were highly stringent, as DS and DP samples, for which the validation protocol is intended to be used, contained only minute levels of these impurities owing to the extensive downstream cleaning procedures.

### 3.2. Linearity

Linearity was demonstrated for the DP_low sample in nine independent plaque assays. Each assay was designed to test six replications (wells) per three 2-fold dilutions for a tested sample, which would give rise to 15, 30, and 60 PFU per well. This was achieved by designating one 6-well plate for each dilution (a total of three plates per assay). Three aspects of linearity were tested: (1) the regression lines, (2) the dependence of precision on dilution, and (3) the dependence of accuracy on dilution.

#### 3.2.1. Investigation of Linearity Using Regression Lines

**Figure 2:**
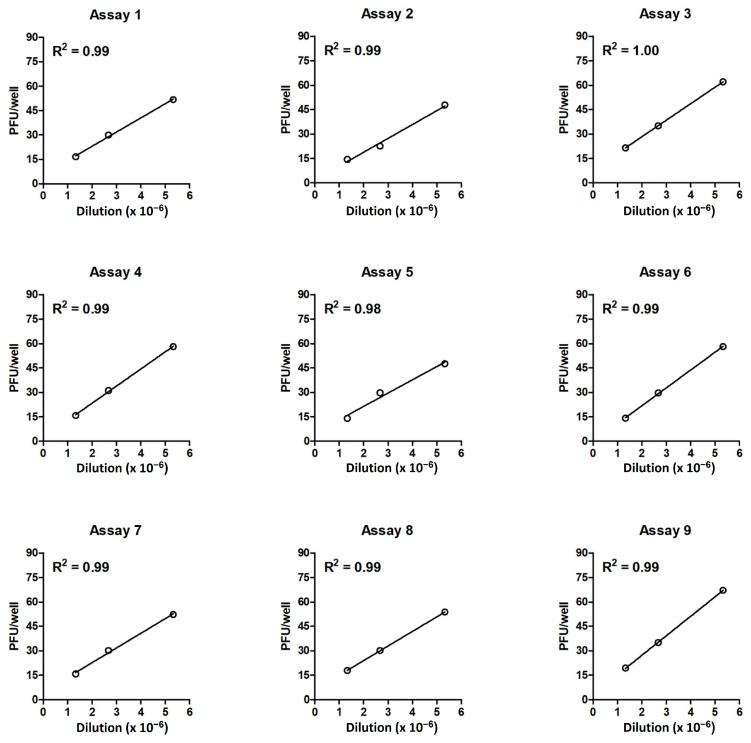
Regression lines for PFU/well results versus dilutions of the DP_low sample in nine independent assays. The x-axis denotes the reciprocal of the dilution factor. The value of each PFU/well (open circle) is the mean of 18 replications (3 independent samples, each tested in 6 wells per dilution). The goodness of fit was calculated via the least squares method.

The linearity was first tested by calculating the goodness of fit (R^2^) of the regression line for PFU/well versus the dilution via the least squares method. The PFU/well value was the mean result of 18 replications: three independent samples, each tested in six wells per dilution. In all nine independent assays, the goodness of fit was found to be very high (R^2^ ≥ 0.98; Figure 2), confirming that the analytical procedure was linear.

#### 3.2.2. Investigation of Linearity Based on Dependence of Precision on Dilution

**Figure 3:**
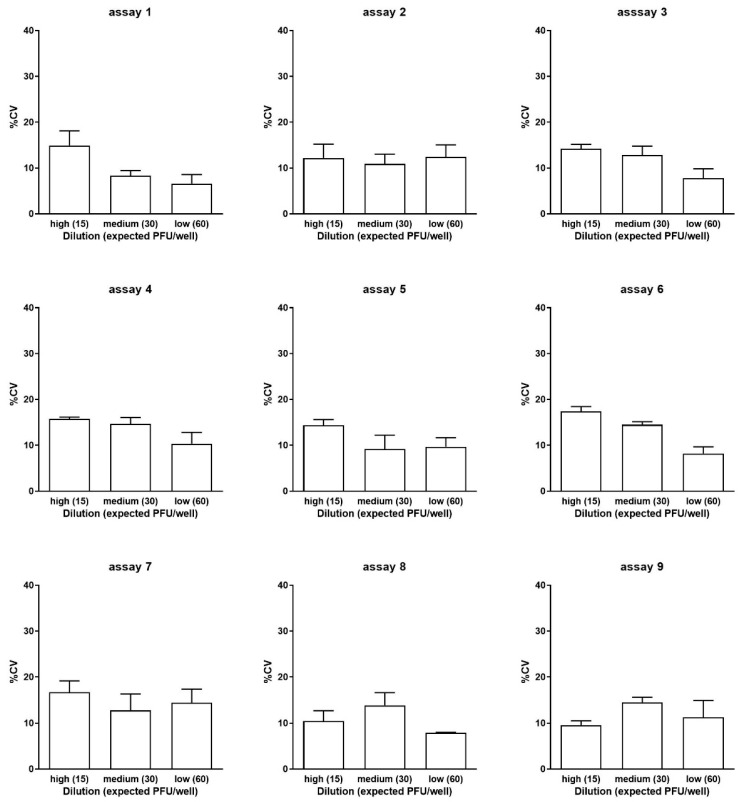
Precision within dilutions. The DP_low sample was tested in three twofold dilutions, giving rise to approximately 15, 30, and 60 plaques/well in nine independent assays. The distribution of the %CV of the plaques in each of the six wells (replicates) for a given dilution is shown. Each column represents the mean ± 1 standard deviation.

The precision within each dilution was tested by calculating the coefficient of variation (%CV) of the plaque number in each of the six wells (replicates) for a given dilution. In this regard, %CV is the proportion of the standard deviation to the mean in terms of percentages. The mean value of the three %CV results from three different vials is shown in Figure 3. Only one out of nine assays (assay 6) revealed a statistical difference among the %CV values. In this specific assay, the %CV of the lowest dilution (the highest number of plaques per well) was lower than the other two %CV values. Nevertheless, all the %CV results were found to be less than 20% in all the assays.

#### 3.2.3. Investigation of Linearity Based on Dependence on Dilution Accuracy

**Figure 4:**
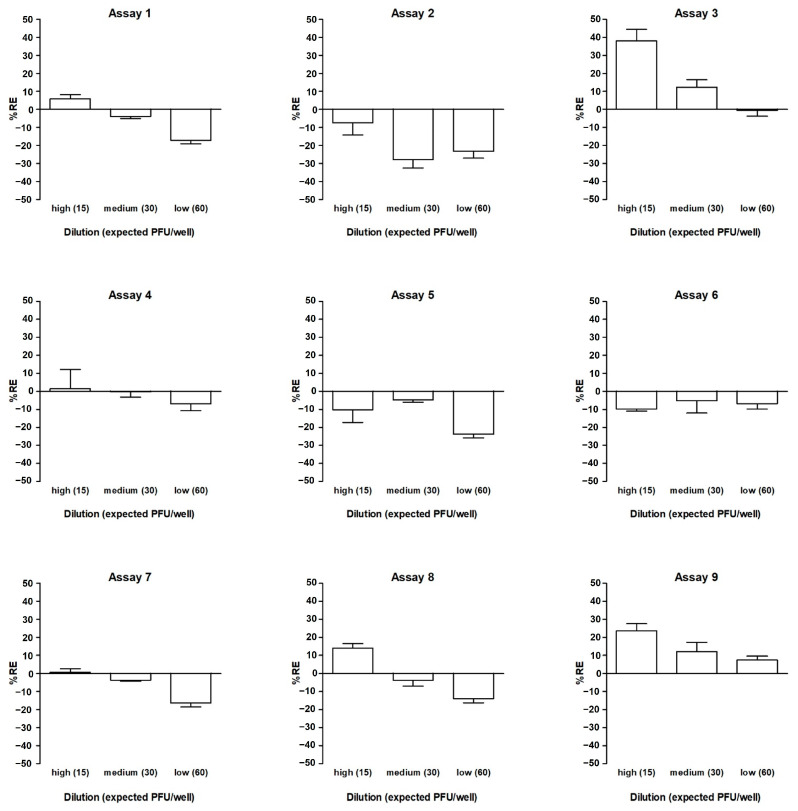
Accuracy within dilutions. Three twofold dilutions of the DP_low sample were analyzed in nine independent assays. The distribution of the %RE calculated from the mean plaque number in six wells for a given dilution is shown. Each column represents the mean %RE derived from three independent vials + 1 standard deviation.

To test the linearity of the results, the accuracy of each dilution was evaluated. The calculation of the relative error (%RE) of the mean plaque number in six wells for a given dilution was performed by subtracting the expected (known) plaque number (according to the predetermined titer) from the mean counted number and dividing by the known plaque number. As three vials were tested per assay, the mean value of the three %RE results is shown in Figure 4. On average, the %RE values were 6.2%, −2.8%, and −11.3% for the high, middle, and low dilutions, respectively.

### 3.3. Range

The linear range was derived from the regression line, precision, and accuracy between the high dilution, which led to the expected 15 PFU/well, and the low dilution, which was expected to give rise to 60 PFU/well. Between 15 and 60 PFU/well, the precision and accuracy were within the accepted criteria for this type of biological assay, with %CVs and %REs of less than 16%, on average, and excellent goodness of fit was recorded, i.e., R^2^ ≥ 0.98; in most of the assays, R^2^ exceeded 0.99. Considering these findings, the range was determined between dilutions that would end in 15–60 PFU/well. However, in practice, the following factors dictate an assay design permitting a maximum of 80 PFU/well for low dilution and not less than 10 PFU/well for high dilution. Firstly, regarding precision, the average %CV was approximately 15%, meaning that two standard deviations would give rise to approximately 30% of the expected PFU/well value; thus, aiming for 60 PFU might result in 80 PFU (high end), while diluting to 15 PFU may result in 10 PFU (low end). Secondly, in addition to the previous point, the lower limit of quantitation (LLOQ) was set to 10 PFU/well, as each plaque counted was less than 10%. In this regard, the presence of as little as one plaque could be distinguished compared to areas of discoloration or other imperfections in the cell monolayer, setting the limit of detection (LOD) as one PFU/well. However, a concentration of less than 10 PFU/well is undesirable. Thirdly, the diameter of the rVSV-SARS-CoV-2-S plaques in the current study limited the plaque number to 80 PFU/well, as more plaques in the well (6-well plate, 35 cm^2^ per well) led to the convergence of plaques, resulting in the inability to delineate different plaques, thus hampering counting.

### 3.4. Accuracy

**Figure 5:**
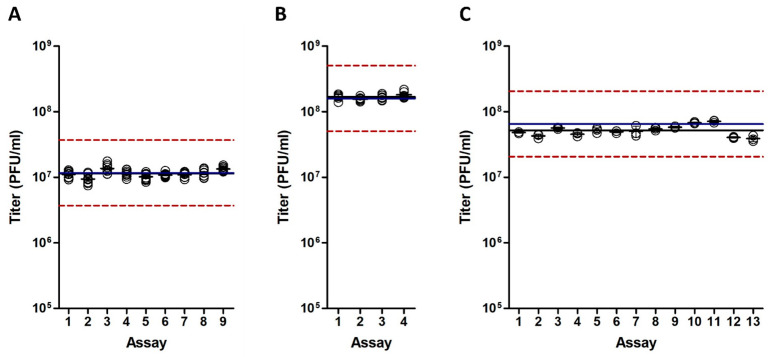
Titer distribution. The viral titers of the three tested samples are shown for each of the validation assays. (**A**). DP_low sample. (**B**). DP_high sample. (**C**). QC sample. In the assays of the DP_low and DP_high samples, each circle represents the mean of nine results obtained from three dilutions within three different vials. The nine results are presented as the means of six wells (repeats). In the assays of the QC sample, each circle is the mean of three results obtained from one dilution within three different vials, where each of these three results is the mean of six wells. The dashed red lines are the known means ± 0.5 log_10_ PFU/mL, the blue lines are the known means, and the black lines are the calculated means.

To determine the accuracy, i.e., the closeness of agreement between the expected virus titer and the measured value, the PFU/mL results obtained in each assay were compared to the expected results according to the known predetermined titer from prevalidation assays. For the QC sample, the titer in each assay was calculated from the mean of 18 results from three different vials, each tested in a 6-well plate (one dilution). For both the tested DP samples, the assay titer was calculated from the mean value of nine results, corresponding to the mean titer within nine 6-well plates, of which three vials were tested in three dilutions. The titer distribution for each tested sample is depicted in Figure 5. Comparing the log_10_ titers enabled us to calculate the deviations (Table 1).

All deviation results met the acceptance criterion within ±0.5 log_10_ PFU/mL from the known value. Moreover, the average measured titers of the DP_low, DP_high, and QC samples were very close to the known titers, as the mean deviations were only 0.01, 0.02, and 0.09 log_10_ titers, respectively.

### 3.5. Precision

The precision of the plaque assay was analyzed to determine the degree of scatter between a series of measurements obtained from multiple samples of the same homogeneous QC samples and vaccine preparations. Precision, resolved as repeatability and intermediate precision, was expressed as the coefficient of variation in the percentages (%CV) of a series of titer measurements (PFU/mL calculation following the multiplication of PFU/well by the dilution factor).

#### 3.5.1. Repeatability

Repeatability, also termed intra-assay precision, expresses precision under the same operating conditions over a short interval of time [12]. The repeatability of the plaque assay was determined for the QC sample and the two DP samples by both the %CV and the 95% confidence interval (CI) (Table 2, Table 3 and Table 4). For the QC sample, the %CV and the 95% CI were obtained from the mean titer and the standard deviation in each assay based on titers from three precalculated results derived from three different vials, each tested in a 6-well plate (a single dilution), as shown in Table 2. For the DP samples (Table 3 and Table 4), the mean intra-assay titer and standard deviation, used to determine the %CV and 95% CI, were calculated from the mean of nine precalculated results within nine 6-well plates (three vials were tested in three dilutions).

The assay repeatability for the QC and DP samples met the acceptance criteria. The %CV values for the tested samples in all the assays were less than 16% (except for assay 7, in which the %CV was 19.6%). Additionally, the 95% CI values were within the limits detailed by the European Pharmacopeia guidelines for vaccinia [11], i.e., ±0.5 log_10_ PFU/mL. In all the assays, the difference between the log_10_ of the titer and the log_10_ of either the upper or lower limit was less than 0.5.

#### 3.5.2. Intermediate Precision

**Figure 6:**
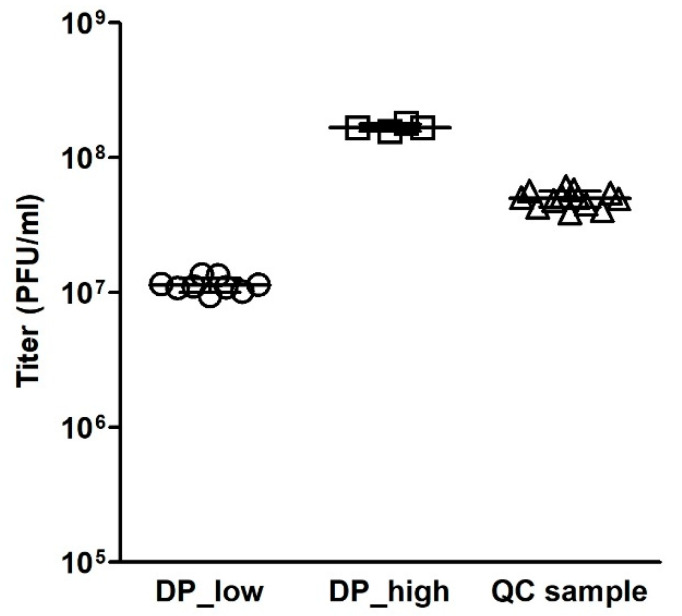
Intermediate precision. For the DP_low sample (circles), variability analysis between the results on four different days and with three different analysts (in a total of nine independent assays) is shown. For the DP_high sample (squares), two different days and two different analysts (in four independent assays) were compared. Additionally, the variability between the titers of the QC samples (triangles) obtained on six different days by three analysts (altogether 13 independent assays) is presented. The mean titers and SDs are given for each sample.

Intermediate precision is a measure of interassay variation. The factors to be considered are potential sources of variability, such as experimental days and different analysts [12]. To assess the intermediate precision, measurements of the DP_low sample made on four different days by three different analysts (altogether nine independent assays) were compared, and measurements of the DP_high sample made on two different days by two different analysts (altogether four independent assays) were also compared. Additionally, as part of the assay control, measurements of the QC sample made on six different days by three different analysts (altogether 13 independent assays) were also compared (Figure 6). The mean titers and %CV values of the DP_low, DP_high, and QC samples were 1.14 × 10^7^ PFU/mL and 12.0%, 1.68 × 10^8^ PFU/mL and 6.0%, and 5.20 × 10^7^ PFU/mL and 18.7%, respectively. Hence, the variability between assays was found to be low, especially when considering both different days and different analysts.

### 3.6. Robustness

**Figure 7:**
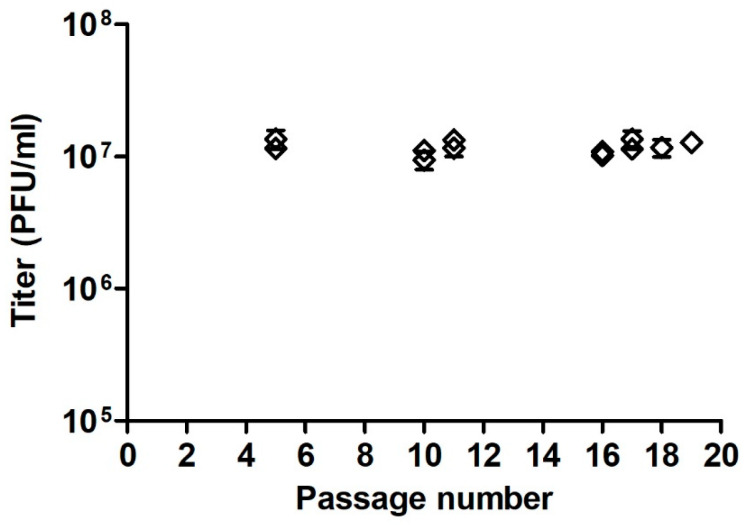
The robustness of the plaque assay. The mean titers ± SDs of the DP_low samples were calculated and are plotted in order of the passage number of the Vero E6 cells.

The robustness of the plaque assay to variations in Vero E6 cell passage was tested to determine the reliability of the method during normal usage. This was carried out by comparing the titers of the DP_low sample to the established value and determining whether titers obtained using a certain cell passage differed from the known value. Differences were defined as those for which the value was greater than 0.5 log_10_ PFU/mL from the established titer. The passage numbers tested were early (5), intermediate (10–11), and late (16–19). After all the cell passages, the titers did not deviate by more than 0.095 log_10_ PFU/mL from the established value. Additionally, the evaluation of the plaque assay’s robustness included correlation analysis between the titer results of the DP_low sample and the cell passage number, ranging from 5 to 19, in 13 assays. Figure 7 shows that no correlation could be observed, as the correlation coefficient r equaled −0.08 (*p* = 0.79).

## 4. Discussion

Titer determination is a basic regulatory requirement for a virus-containing vaccine since it provides a reproducible and accurate production process and, most importantly, reflects vaccine potency. Notably, the vaccine titer is a quantitative measure of the active content, but to serve as a potency test, the titer must correlate with the in vivo results [13]. The determination of the virus titer via the plaque assay was performed during preclinical trials, including the good laboratory practice (GLP) toxicity study performed in rabbits. In accordance with the IIBR BriLife^®^ vaccine clinical trials in Israel, vaccine doses were determined according to the validated plaque assay results. Indeed, human sera from BriLife^®^ vaccinees overall maintained a neutralizing antibody response against all tested SARS-CoV-2 variants, as was recently measured using a plaque reduction neutralization test (PRNT) [14]. Taken together, the newly validated plaque assay can be regarded as a potency-indicating assay, reflecting the anticipated immune response to the vaccine.

The virus concentration determined using the plaque assay should be monitored from the earliest production stage (i.e., at the time of virus harvest from the bioreactor) and throughout the whole production process to monitor the process precision and batch-to-batch consistency and determine the yield during the production stages. Nevertheless, DS and DP samples are the most important from the patient’s point of view; therefore, the plaque assay should be fully validated at least for those samples, as described in the current report. This report summarizes the validation results for the DS and DP vaccine preparations.

A plaque assay was used to determine the viral titers of live viruses in various viral vaccines [15,16,17,18,19], including the smallpox vaccine previously produced at the IIBR GMP facility and via the PRNT-related method [20]. Plaque assays are the most appropriate method for determining the concentration of live virus and reflecting potency since their results, reported in PFU/mL, indicate the concentration of infective particles within the sample. However, plaque assays may have certain limitations. Plaque assays are only applicable to viruses that form countable plaques. Additionally, they are labor-intensive and time consuming and require highly trained analysts to prevent potential subjective interpretation of the results. Given technical advancements in recent years, attempts are being made to develop novel approaches to address some of the plaque assay limitations. For instance, the rapid in-process measurement of live virus vaccine potency using label-free laser force cytology was recently reported [21]. The authors were able to measure the potency of the measles virus in upstream biomanufacturing process in real time and compare this to the traditional tissue culture infectious dose 50% (TCID_50_) potency. Another recent study described a flow virometry assay capable of rapidly detecting damaged virus particles for the process monitoring of the ERVEBO live-virus vaccine. The results correlated with reduced infectivity [22]. However, alternative strategies for replacing the standard plaque assay remain premature, and for the time being, this is the only pharmacopeial test that is suitable for measuring live virus vaccines. Accordingly, the validation of a plaque assay designed to determine the viral titer of a vaccine is crucial. Shurtleff et al. were the first to validate a quantitative plaque assay for use in preclinical studies, following the validation characteristics required by the U.S. Pharmacopeia [23]. The authors showed that the plaque assays were accurate, precise, and robust for filovirus titration. They relied on the Bioanalytical Method Validation Guidance for Industry of the United States Food and Drug Administration (FDA), and the acceptance criteria were determined from the chapter on the validation of alternative microbiological methods of the U.S. Pharmacopeia (USP) [24]. The validation presented herein relies on the requirements of the European Pharmacopoeia (EP) for PFU determination for the smallpox vaccine (live) [11].

Until now, neither the pharmacopoeia nor other regulatory documents have published any acceptance criteria regarding the validity of determining the concentration of live virus in a vaccine produced specifically from the SARS family of viruses. Therefore, the current validation was based on the pharmacopeial requirements specified in the chapter discussing the validity of the PFU test for smallpox vaccines within the European Pharmacopoeia [11]. Thus, the validation protocol followed the criteria and requirements of the ICH guidelines discussing the validation of analytical methods [12], ultimately demonstrating that the test is indeed suitable for use in its intended purpose.

The plaque assay is not considered a classic analytical test, as is ELISA; therefore, some adjustments to the regulatory directives were needed. For example, specificity was proven in each batch by spike sequencing using the PCR method, as well as through complete virus sequencing using NGS. In addition, the presence of other viruses and adventitious agents was excluded in every production batch via transmission electron microscopy (TEM) and a series of tests performed at Charles River laboratories, including in vitro tests for bovine adventitious viral agents in several cell lines.

The first step in the validation process was to demonstrate the linearity and linear range of the data. To show that the results were linear throughout the test range, i.e., between the lower (10 PFU/well) and the upper (80 PFU/well) threshold, the range had to be determined based on the precision and accuracy of the PFU results deduced from the tested dilutions and via linear regression between PFU values and dilutions, designed to give rise to 15, 30, and 60 PFU/well on average. Within this range, the precision of all dilutions was acceptable (CV < 16%, on average), the accuracy of all dilutions was acceptable (RE < 15%, on average), and the goodness of fit of the linear regression of the PFU/well versus dilution was acceptable (R^2^ ≥ 0.98). In light of these results, each batch was tested in three dilutions, in which the high dilution led to an average of 15 PFU/well, and the low dilution led to an average of 60 PFU/well. However, in practice, and in accordance with the expected variability, this range allows us to count 10 PFU/well at high dilution and 80 PFU/well at low dilution. Taken together, the statistical analysis revealed that the precision, accuracy, and linearity of the data met the criteria needed for an analytical test throughout the working range between dilutions aimed at receiving 15–60 PFU/well and, therefore, included a lower threshold for counting 10 PFU/well (LLOQ) and an upper threshold for counting 80 PFU/well.

The accuracy of the plaque assay was demonstrated by calculating the difference between the log_10_ of the measured titer and the log_10_ of the known titer for the QC and DP samples, which had been determined via the prevalidation assays. These were plaque assays conducted in the same way as the validation assays, i.e., using six-well plates with Vero E6 monolayers inoculated with VSV-SARS-CoV-2-S samples. As required by health regulators, the prevalidation assays were inherent to the validation protocol and carefully planned and executed. The validation process continued only after achieving absolute control over the prevalidation results and accumulating reliable and comprehensive data on all aspects of the plaque assay, including the titers of the various samples.

According to the European Pharmacopeia directives, the acceptable concentration of the reference preparation in this complex biological assay should not differ by more than 0.5 log_10_ PFU/mL from the established value [11]. The accuracy of the results was within these limits, as the maximal differences between the measured and known log_10_ titers of the DP_low, DP_high, and QC samples were 0.09, 0.05, and 0.22, respectively. Nevertheless, as shown, on average, the validation presented much lower deviations, as the mean differences were as low as 0.01, 0.02, and 0.09 for the DP_low, DP_high, and QC samples, respectively. It should be noted that the fact that some of the deviations were upward and some were downward from the expected value could eliminate any permanent bias in a certain direction.

The repeatability of the plaque assay was also determined for the QC sample and the two DP samples by both the %CV and 95% CI. According to the European Pharmacopeia, the 95% CI of the virus concentration and the reference preparation should be less than ±0.5 log_10_ PFU/mL [11]. In all the tested samples, the 95% CI values met these criteria, with a maximal value of 0.28 log_10_ PFU/mL for the QC sample in 1 out of 13 assays. Moreover, in all the other assays, including those for the DP_low and DP_high samples, the 95% CI was 0.1 log_10_ PFU/mL or much lower. In addition, the intra-assay precision in all the tested samples met the analytical assay criterion of ICH guidelines [12], as none of the calculated %CV values exceeded 16%. In light of these low 95% CI and %CV values and despite the biological complexity of the plaque assay, the validation process demonstrated high repeatability. In addition, the intermediate precision, i.e., the interassay variability, was also low within the limits of an analytical assay, as %CV values between plaque assays for all samples were low and analytically acceptable, being equal to 18.7%, 12.0%, and 6.0% for the QC, DP_low, and DP_high samples, respectively. Taken together, these results demonstrated both high repeatability and intermediate precision, demonstrating that the presented plaque assay can be used for its intended purpose.

One of the challenges that must be addressed in the validation of an analytical method is the method’s robustness. This was achieved by making an initial change to verify that the test was immune to this change. The parameter that was tested herein was the number of cell passages between the early passage (5), intermediate passages (10–11), and late passages (16–19). The acceptance criterion was that the sample titer be within the limits of 0.25 log_10_ PFU/mL, which is the difference from the established value. Indeed, the data demonstrated that regardless of the passage number of the cells used in the assay, the titers obtained deviated from the established, predetermined titer of the DP_low sample by only 0.095 log_10_ PFU/mL or less. Additionally, as evidenced by the correlation coefficient calculated (r = −0.08, *p* = 0.79), the virus titer did not depend on cell passage.

In conclusion, the newly developed plaque assay meets all the stringent pharmacopeial requirements of a bioanalytical method and is suitable for determining the concentrations of live rVSV-SARS-CoV-2-S virus in the tested DP samples of the BriLife^®^ vaccine. Furthermore, the validation protocol presented herein may be a reliable methodology for validating plaque assays for other live virus-based vaccines.

## Figures and Tables

**Table 1:** Deviations of measured titers from known titers.

QC Sample		DP_Low		DP_High	
Assay	Deviation ^1^	Assay	Deviation	Assay	Deviation
1	0.13	1	0.02		
2	0.18	2	0.09		
3	0.06	3	0.07		
4	0.16	4	0.01		
5	0.10	5	0.06		
6	0.12	6	0.03		
7	0.11	7	0.03		
8	0.08	8	0.01		
9	0.05	9	0.06		
10	0.02			1	0.02
11	0.04			2	0.01
12	0.20			3	0.02
13	0.22			4	0.05

^1^ Deviations are absolute values of the log_10_ titer differences.

**Table 2:** The repeatability of the QC sample titers.

Assay	Mean Titer (×10^7^ PFU/mL)	Mean Titer (Log_10_)	Lower 95% CI		Upper 95% CI		%CV
			Min.	Length ^1^	Max.	Length	
1	4.82	7.68	7.65	−0.03	7.71	+0.03	12.6%
2	4.28	7.63	7.60	−0.03	7.66	+0.03	14.3%
3	5.65	7.75	7.72	−0.03	7.78	+0.03	12.6%
4	4.54	7.66	7.62	−0.03	7.69	+0.03	14.7%
5	5.21	7.72	7.68	−0.04	7.75	+0.03	15.9%
6	4.95	7.69	7.66	−0.03	7.72	+0.03	13.6%
7	5.05	7.70	7.66	−0.04	7.74	+0.04	19.6%
8	5.46	7.74	7.72	−0.02	7.76	+0.02	9.3%
9	5.81	7.76	7.74	−0.02	7.79	+0.02	9.8%
10	6.78	7.83	7.81	−0.02	7.85	+0.02	7.8%
11	7.13	7.85	7.82	−0.03	7.88	+0.03	14.3%
12	4.06	7.61	7.58	−0.02	7.63	+0.02	11.1%
13	3.90	7.59	7.56	−0.03	7.62	+0.03	14.5%

^1^ Length is the difference between either the lower 95% CI (Min.) or the upper 95% CI (Max.) and the log_10_ titer.

**Table 3:** The repeatability of the DP_low titers.

Assay	Mean Titer (×10^7^ PFU/mL)	Mean Titer (Log_10_)	Lower 95% CI		Upper 95% CI		%CV
			Min.	Length	Max.	Length	
1	1.11	7.05	7.01	−0.04	7.08	+0.04	10.9%
2	0.94	6.97	6.92	−0.05	7.02	+0.05	15.2%
3	1.36	7.13	7.08	−0.06	7.18	+0.05	15.8%
4	1.15	7.06	7.02	−0.04	7.10	+0.04	11.1%
5	1.02	7.01	6.97	−0.04	7.05	+0.04	12.2%
6	1.08	7.04	7.01	−0.03	7.06	+0.02	7.4%
7	1.09	7.04	7.01	−0.03	7.07	+0.03	8.6%
8	1.16	7.06	7.02	−0.05	7.10	+0.04	13.1%
9	1.34	7.13	7.10	−0.03	7.15	+0.03	8.1%

**Table 4:** The repeatability of the DP_high titers.

Assay	Mean Titer (×10^7^ PFU/mL)	Mean Titer (Log_10_)	Lower 95% CI		Upper 95% CI		%CV
			Min.	Length	Max.	Length	
1	16.6	8.22	8.18	−0.05	8.26	+0.04	9.4%
2	15.7	8.20	8.16	−0.03	8.23	+0.03	6.9%
3	16.6	8.22	8.19	−0.03	8.25	+0.03	7.9%
4	18.1	8.26	8.22	−0.04	8.29	+0.03	10.9%

## Data Availability

The data presented in this study are available upon request from the corresponding author.
